# Improving drought tolerance in maize: Tools and techniques

**DOI:** 10.3389/fgene.2022.1001001

**Published:** 2022-10-28

**Authors:** Michael S. McMillen, Anthony A. Mahama, Julia Sibiya, Thomas Lübberstedt, Walter P. Suza

**Affiliations:** ^1^ Department of Agronomy, Iowa State University, Ames, IA, United States; ^2^ School of Agricultural, Earth and Environmental Sciences, University of KwaZulu-Natal, Pietermaritzburg, South Africa

**Keywords:** drought tolerance, food security, maize breeding, genomics assisted selection, genome mapping, model-assisted approaches, plant breeding education

## Abstract

Drought is an important constraint to agricultural productivity worldwide and is expected to worsen with climate change. To assist farmers, especially in sub-Saharan Africa (SSA), to adapt to climate change, continuous generation of stress-tolerant and farmer-preferred crop varieties, and their adoption by farmers, is critical to curb food insecurity. Maize is the most widely grown staple crop in SSA and plays a significant role in food security. The aim of this review is to present an overview of a broad range of tools and techniques used to improve drought tolerance in maize. We also present a summary of progress in breeding for maize drought tolerance, while incorporating research findings from disciplines such as physiology, molecular biology, and systems modeling. The review is expected to complement existing knowledge about breeding maize for climate resilience. Collaborative maize drought tolerance breeding projects in SSA emphasize the value of public-private partnerships in increasing access to genomic techniques and useful transgenes. To sustain the impact of maize drought tolerance projects in SSA, there must be complementary efforts to train the next generation of plant breeders and crop scientists.

## Introduction

Maize is the primary staple food for more than 900 million people globally and the third most important source of calories after rice and wheat (Shiferaw et al., 2011; Adebayo and Menkir, 2015). With a decrease in rice production in China and India, and an increase in the demand for dairy and meat, global demand for maize is projected to double by 2050 (Rosegrant et al., 2009). Maize is also the most widely grown staple crop in SSA and serves an important role in food security, but it is also highly susceptible to drought, with 15%–20% of its yield lost to drought each year (FAOSTAT, 2010; Bankole et al., 2017; Lunduka et al., 2017; FAO, 2021). Because of maize yield loss to drought, from 2005 to 2915, developing countries experienced a revenue loss of up to USD 29 billion (FAOSTAT, 2010; Liu and Qin, 2021). Changes in rainfall magnitude, distribution, and timing, as well as an increase in temperature, all interact to destabilize maize production further (IPCC, 2012; Meseka et al., 2018). Data from more than 20,000 trials in Africa revealed a 1% reduction in maize yield for each “degree day” above 30°C (Lobell et al., 2011; Bankole et al., 2017; Lunduka et al., 2017). When other factors are not limiting, the combination of heat and drought stress causes a 1.7% yield reduction for each excess degree day (Lobell et al., 2011).

An assessment of the impact of physical drought on maize revealed an alarming vulnerability in SSA (Kamali et al., 2018). On average, 5 to 10 drought events were experienced between 1970 and 2004 in most parts of SSA (Fisher et al., 2015). This makes drought a key constraint to maize production in the region (Heisey and Edmeades, 1999). Further, climate change is expected to exacerbate the impact of drought in the region and cause a reduction in maize production by almost 22% in 2050 (Schlenker and Lobell, 2010; Challinor et al., 2016; Tesfaye et al., 2018; Barbosa et al., 2021). Therefore, meeting the increased demand while stabilizing production requires a strategy that includes the genetic improvement of maize for drought tolerance (Messina et al., 2021). A recent review by Sheoran et al. (2022) discussed new breeding technologies and approaches to improve maize drought tolerance. This review presents an overview of a broad range of tools and techniques, the integration of plant physiology, molecular biology, and systems modeling, and examples of private-public partnerships in developing drought tolerant maize for Africa. The review also discusses the importance of plant breeding education to address the shortage of plant breeders in SSA.

## Breeding for drought tolerance in maize

Drought is a key abiotic stress that causes low-income countries to lose billions of dollars (FAO, 2021). Levitt (1972) provided functional definitions for water deficit and drought stress which can be used to develop breeding targets. Water deficit occurs when plant transpiration cannot fully meet the atmospheric demand due to a lack of water in the environment. This deficit causes damage and induces a stress response proportional to the rate of deficit (Blum, 2014).

The impact of drought stress depends on the interaction of plant, environmental, and management factors: the crop development stage, rate of water deficit development, peak intensity of the deficit, and planting density. Regarding the development stage, maize is most sensitive to drought stress during flowering (Bolanos and Edmeades, 1993). Severe water deficits during the period of a few days before silking to roughly 25 days can eliminate yield entirely (Claassen and Shaw, 1970). Drought stress causes a delay in ear growth and silking, increasing the anthesis-silking interval (ASI) to the point where it inhibits fertilization. The result is a barren ear or one with few kernels (Sah et al., 2020). Even with successful pollination, kernel abortion beginning as early as 2–3 days after pollination can reduce kernel number (Westgate and Boyer, 1986). Drought stress at the start of grain filling can also significantly lower or eliminate yield (Barker et al., 2005). Since the crop reaches full size prior to flowering, water use is at a maximum. Drought stress induces premature leaf senescence and reduces ear growth, with severe stress causing complete desiccation. Consequently, kernel weight is significantly reduced due to lowered photosynthate accumulation.

According to Ribaut et al. (2009), maize responds to and mitigates the impact of water deficit using three primary strategies: drought escape, drought avoidance, and drought tolerance. Drought escape is a strategy to prevent the coincidence of water deficit with key developmental stages and is primarily achieved by early flowering and maturation. Drought avoidance, on the other hand, is the capacity to avoid or reduce plant water deficit but maintaining turgor through an increase in water uptake (using a deeper and/or a larger root system for example) and/or a reduction in water use (for instance, decreased stomatal conductance). Drought tolerance is the ability to maintain plant function during water deficit, which can be achieved by alleviating oxidative stress, for instance.

The goal of a drought tolerance breeding program is two-fold: 1) reduce the gap between yields in optimal and stress conditions without sacrificing yield potential, and 2) improve yield stability for a range of stress conditions. According to Lunduka et al. (2017), a drought tolerant maize variety is one that gives at least a yield of 30% of its potential under water-stress, especially during the flowering and grain-filling stages. In addition, Messina et al. (2021) found that although root systems architecture and yield have changed because of breeding for maize drought tolerance, the uptake of water has not changed.

While drought escape does create an advantage under drought conditions, using earlier maturing maize varieties which generally yield less than full season varieties (White et al., 1922) in non-drought years creates a lower yield potential (Ke and Ma, 2021). To improve both yield potential and stability, the use of hybrids with a maturity suited to the wettest part of the year in the target environment might be an option. Together, the capacity of a plant to avoid or reduce water deficit (dehydration avoidance), sustain function under water deficit (dehydration tolerance), improve crop productivity and provide avenues for plant improvement.

Even without drought tolerance as an explicit breeding goal, selecting for high yield potential under well-watered conditions has consistently led to increased yield in both water deficit and non-deficient environments (Castleberry et al., 1984). Selecting for high yield potential extends tolerance to other abiotic stresses as well, such as heat, cold, and low soil fertility. The improvement in drought tolerance and the rate of genetic gain has been evaluated through experiments testing historic cultivars under drought conditions applied at various growth stages, demonstrating a gain of 124 kg ha^−1^ yr^−1^ for flowering stress and 91 kg ha^−1^ yr^−1^ for mid grain fill stress (Barker et al., 2005). Nevertheless, the ability of newer hybrids to tolerate drought stress is primarily due to the adaptation of parent lines to higher planting densities (Tollenaar and Lee, 2006).

Although selecting for high yield potential in favorable environments has led to increases in drought tolerance, the correlation of yield for hybrids grown under well-watered and water-stressed conditions is reduced with the degree of stress. In addition, the degree of genotype by environment interaction necessitates screening materials under both well-watered and water stressed conditions. Therefore, because drought is unpredictable in the target environment, sites in rain-free environments, which use irrigation and planting date to control the timing and severity of drought, are needed (Campos et al., 2004).

### Choosing testing environments

The rate of achievable genetic gain is highly dependent on the choice of testing environments, especially how closely the selection environments mirror the target environments (Cooper et al., 2006). Since the distribution and amount of rainfall, temperature, soil water holding capacity, and the developmental stage of the crop all interact to create distinct drought scenarios, an important preliminary step in developing a breeding program for drought tolerance is capturing this information to identify the target population of environments (TPE) for which the crop will be adapted (Cooper et al., 1997).

Environmental characterization and TPE delineation are crucial for several reasons. Information on the TPE can be used to explain and predict a considerable portion of genotype by environment (G x E) interactions. This is necessary since the effect of a particular allele can be different depending on environmental conditions (Chenu et al., 2009). In one drought scenario a trait can be advantageous, while in others it can be detrimental. For instance, breeding for water use efficiency can improve yield in very dry environments, while reducing the potential yield in mild drought conditions (Tardieu, 2012).

Characterizing the TPE is also crucial in identifying appropriate managed stress environments for multi-environment trials (METs) since the expected performance gain is dependent on the similarity between environments represented in METs and the TPE (Ribaut et al., 2009). Furthermore, by weighting phenotype data from METs based on how representative individual trials of the TPE are, selection gain over generations can be improved. This improvement in gain was demonstrated by Podlich and Cooper (1998) using a genetic simulation model. Comparing the drought scenarios of the TPE with other drought-prone regions facilitates methodological and adapted germplasm exchange to other parts of the world (Chenu et al., 2011). Increases in temperature and changes in weather patterns brought about by climate change highlight the need to characterize the target environment in advance, since the environmental conditions could be significantly different by the time a variety is developed and disseminated, resulting in lower-than-expected yields (Challinor et al., 2016). These seasonal variability and resource constraints often lead to multi-environment breeding trials offering biased representation of the TPE. One way to avoid this is use of weighted analysis based on representative trials which can help breeders select for germplasm better adapted to the TPE. Managed-environment trials are another way to evaluate performance in representative environments or for stresses, allowing detailed assessment of germplasm, traits, or genes of interest (Chenu, 2015).

As mentioned above, crop simulation models have been used to characterize TPEs, and to evaluate how well MET locations fit the TPE. Studies using these models help to improve breeding efficiency for multiple crops in regions around the world. For instance, the “*Cerrado*” environments of central Brazil have been characterized for rice and maize production (Heinemann et al., 2008). More recently, maize growing environments in Eastern and Southern Africa were characterized using the APSIM model, leading to the distinction of four environmental types to which breeding objectives can be catered (Seyoum et al., 2017).

In addition to similarity to the target environment, site homogeneity and the ability to manage water inputs determine the success of testing (Blum, 2011b). Differences in soil texture, effective rooting depth, micronutrient concentration, salinity, pH, and the presence of pathogens all increase residual variability, thus minimizing the precision in estimating genotypic means. Regarding the ability to manage water inputs, use of desert and off-season environments, and rainout shelters provide options to prevent the effect of precipitation (Blum, 2011b). One issue with the use of desert environments is the effect of temperature extremes. Estimating drought tolerance is confounded by the occurrence of extreme temperatures during ear growth (Otegui and Andrade, 2000). Recent improvements in modeling techniques have increased their effectiveness. Liu et al. (2021) concluded that DSSAT CERES-Maize can adequately simulate regional maize yields using the CERES-Maize module calibrated to regional soil and daily weather databases. Adnan et al. (2020) successfully use the CERES-Maize model to generate data for GEI and stability studies of maize genotype in the absence of observed field data, and Ramirez-Villegas et al. (2020) pointed out the varying important roles of crop modeling in breeding efforts, including assessing genotypic adaptability and stability, characterizing and identifying target breeding environments, identifying tradeoffs among traits for such environments, and making predictions of the likely breeding value of the genotypes. Recognizing the successes from simulation modeling, Hajjapoor et al. (2022) pointed out the problems that still exist in identifying MET environments that fit TPEs, to deal with Genotype-by-Environment-by-Management (G x E x M) interactions and proposed a simple step-by-step approach to bring the capacity of process-based models to better define target population of environment, within which the clustering of subunits will allow for the reduction of G x E × M interactions.

### Incorporating secondary traits

Although maximizing grain yield is the primary objective of breeding for improved drought tolerance, reduced genotypic variance and high G × E interaction contribute to reduced heritability of yield when testing under drought conditions. Incorporating secondary traits into the selection process, that is, traits which more directly reflect the physiological effect of drought stress, can increase selection efficiency, and improve gain (Ribaut et al., 2009). Edmeades et al. (1996a) developed criteria for ideal secondary traits for drought screening. In addition to high heritability, high genetic variability, genetic correlation with yield, and no association with yield loss under non-limiting conditions, ideal traits should be simple, cheap, non-destructive, and fast to assay. Using these criteria, only ASI, ears per plant (EPP), barrenness, kernels per ear, and stay green have been found to be suitable secondary traits. Of those traits, ASI and EPP have been identified as the best performing traits, with ASI being the most widely used. While tassel growth is not as affected by drought stress, silk emergence and thus ASI can be used as an indicator for ear and plant growth rates during flowering. When testing under conditions that reduce yield by more than 50%, the incorporation of these secondary traits into a selection index has led to a selection efficiency on a par with testing under optimal conditions (Chapman et al., 1997). Using yield alone for selection resulted in significantly lower gains but the use of secondary traits for selection has resulted in improved genetic gains in other instances as well (Campos et al., 2004).

### Application of genomic mapping tools

While the use of secondary traits provides more heritable selection targets, the advancing field of genomics has been used to further improve selection efficiency by identifying and selecting the genomic regions responsible for improved secondary traits and tolerant phenotypes. By developing a better understanding of the genetic and physiological basis of drought tolerance, and using this information during selection, the value of genomics in improving drought tolerance has been demonstrated in maize (Tuberosa et al., 2007; Tsonev et al., 2009), in addition to several other crops (Tuberosa and Salvi, 2006; Mir et al., 2012). With marker-assisted selection, the initial step is to identify markers and genes associated with drought tolerance. These associations have been identified using a variety of approaches. In addition, the use of parental haplotype sharing can help increase the power, precision, and accuracy in Quantitative Trait Loci (QTL) mapping (Jansen, Jannink, and Beavis, 2003).

Most understanding of the genetic basis of drought tolerance was gained from traditional QTL mapping, using a relatively limited number of markers in bi-parental populations (Cattivelli et al., 2008). While a large number of associations have been found, there are limitations with traditional QTL mapping: 1) the resulting QTL are comparatively large due to limited genetic resolution; 2) extra time is required to develop a mapping population; 3) by using a mapping population, only a small proportion of the total allelic diversity expected in the germplasm pool is sampled; and 4) QTL for the same trait can segregate differently in other mapping populations.

Using linkage mapping, numerous QTL relating to morphological traits such as flowering and tassel size, as well as physiological parameters such as ABA and carbohydrate metabolism have been identified. Several of these QTL studies have been summarized by Ribaut (2006). To identify genome regions and candidate genes consistent across populations, which convey drought tolerance, QTL from various studies have been analyzed and compiled into consensus maps (Tuberosa et al., 2002; Sawkins et al., 2004; Hao et al., 2009; Semagn et al., 2013; Zhao et al., 2018).

Linkage disequilibrium (LD)-based association mapping has been used to overcome some of the constraints of linkage mapping. The advantages of LD association mapping are summarized by Mir et al. (2012) and include: less time and resources are required since a natural germplasm collection can be used, and it provides a higher mapping resolution. LD studies also have the advantage of being able to simultaneously evaluate the varying effects of multiple alleles in multiple backgrounds at one time (Buckler and Thornsberry, 2002). Since diverse germplasm is used for association mapping, identified markers are more likely to convey drought tolerance in multiple backgrounds which is particularly valuable to breeders.

Although linkage and LD-mapping both have unique advantages, the approaches are complementary (Myles et al., 2009) and have been combined to better identify QTL associated with drought tolerance. Using the combined techniques, SNP markers were identified, which better explained the phenotypic variation regarding ASI compared to either technique alone (Lu et al., 2010). However, association mapping is not without limitations. One drawback is the difficulty in detecting associations with traits underpinned by many rare variants with a large effect size, or by many common variants that have a small effect (Korte and Farlow, 2013). Since the effect size of the allelic variants as well as their frequency in the sampling population determine the phenotypic variance, rare variants, and a combination of alleles with small effect sizes are difficult to associate (Korte and Farlow, 2013).

As sequencing technologies have advanced and costs have dropped, genome-wide association studies (GWAS) have become a common approach to uncover the genetic basis of drought tolerance (Yamada and Dwiyanti, 2013). GWAS has been successful in identifying genome regions associated with drought tolerance. Using 60,000 SNPs on a hybrid testcross background allowed dominant alleles to be detected. Farfan et al. (2015) identified 10 quantitative trait variants (QTVs) for flowering time, plant and ear height, and yield. Under both well-watered and stressed conditions, three of these QTVs explained 5–10% of yield variation. Many of the QTVs also co-located with QTL from other studies, which confirmed their association with drought tolerance. Rather than using a high-density coverage of the maize genome, other association studies have taken a more targeted approach and instead have used a comparatively limited number of SNPs (1536) selected from candidate genes associated with drought response (Setter et al., 2011).

While Setter et al. (2011) identified several significant loci and candidate genes, it is possible that many were missed due to a lack of genome coverage. To enlarge the panel of markers for the collection without additional genotyping, Zhang et al. (2016) used imputation based on 556,809 SNPs from 368 diverse inbred lines that were previously genotyped using RNA sequencing. This method resulted in the identification of 26 new loci associated with metabolic and physiological traits in leaf tissue, and only one of the six loci significantly associated with drought-related metabolites from the Setter study (Setter et al., 2011) was still significant. Positional cloning of QTL in conjunction with association mapping helped to identify genes and noncoding sequences associated with flowering time, a trait strongly associated with drought response (Salvi et al., 2007).

Nested Association Mapping (NAM), which relies on a specifically designed population, also combines the advantages of linkage and association mapping while minimizing the disadvantages of each (Yu et al., 2008). By crossing 25 inbred lines to the B73 inbred and selfing the F_2_ populations to the F_6_ generation, 200 recombinant inbred lines (RILs) were created for each of the 25 populations. These RILs were genotyped using the same 1106 markers to identify recombination blocks and the parents were sequenced, resulting in 5000 RILs (known as the US-NAM population) that were genotyped at high density and could be compared and analyzed together (Yu et al., 2008). Researchers from the Institute of Crop Science at the Chinese Academy of Agricultural Sciences used both the US-NAM and China NAM populations to identify 52 candidate genes with differential expression under two water treatments, allowing them to make genomic predictions of drought-related traits with a mean accuracy of 0.57 (Li et al., 2016).

Candidate genes have also been identified by incorporating functional genomics technologies, such as transcriptome and metabolome analysis. These approaches help reveal how biochemical, physiological, and regulatory networks change in response to stress, and expose differences in drought response among tissues and genotypes (Mir et al., 2012). By studying gene expression in pre-fertilization ears using both cDNA microarray and genome-survey technology, Zinselmeier et al. (2002) demonstrated differential gene expression among tissues, and identified several genes not previously associated with drought stress response. Microarray technology also facilitated the identification of 22 differentially expressed genes which co-located on the genetic map with QTL previously associated with drought tolerance (Marino et al., 2008).

Like gene expression analysis, examining changes in the plant metabolome can connect the agronomic phenotype with the underlying genetics, and can be useful in identifying genes that are not as affected by environmental factors (Riedelsheimer et al., 2012). A distinct advantage of profiling the metabolome (and proteome) over the use of transcriptomics is that effects from posttranscriptional and posttranslational regulation can be accounted for (Tuberosa et al., 2007). By examining metabolic changes in maize leaves and ears due to water deficit and dissecting the genetic basis of those traits using GWAS, Zhang et al. (2016) identified 23 metabolite-associated loci and validated 10 as responsive to drought stress. Using the same technique on leaves alone, Riedelsheimer et al. (2012) identified 26 SNPs strongly associated with changes in distinct metabolites, which explained up to 32% of the observed genetic variance.

More exciting research results have been achieved in recent years on the genetics of drought tolerance/resistance. The identification of naturally occurring loci or genes associated with drought tolerance can serve as direct targets for both engineering and selecting improved maize for drought regions. Increased expression of the NAC gene (*ZmNAC111*) with the MITE (significantly associated with natural variation in maize drought tolerance) insertion in its promoter enhanced drought tolerance in maize seedlings (Mao et al., 2015). Wang et al. (2016) reported that transgenic maize with enhanced expression *ZmVPP1* (also with natural variation) exhibited improved seedling drought tolerance. In a study of a NAC-encoding gene of *ZmNAC080308*, a functional marker developed for use in predicting drought stress tolerance in a US maize inbred line panel showed that lines carrying Hap2 produced greater than 10% grain yield than those carrying Hap1 under drought stress condition (Wang et al., 2021). Wang et al. (2022) identified and reported the overexpression of a transcription factor, *ZmERF21*, is tightly associated with drought tolerance in maize seedlings, expressed by significantly increased chlorophyll content and activities of antioxidant enzymes under drought conditions. Another GWAS study (Khan et al., 2022) identified candidate genes and their key variations that will contribute to an understanding of the genetic basis of drought tolerance, especially for the female inflorescence, and will be important in facilitating drought-tolerant maize breeding.

### Bridging the gene-to-phenotype gap

Advances in molecular biology have provided tools and strategies to associate genomic regions with traits that convey improved yield in a drought scenario. With genomic selection, these associations can be used for phenotypic prediction and breeding decisions. However, predicting the effects of genes across scales of biological organization is made difficult by the complex interaction of genes and environmental factors (Hammer et al., 2006). Much of the time the effect of a particular allele on a complex trait such as drought tolerance is confounded by gene interactions such as epistasis and pleiotropy (Hammer et al., 2006). In addition to gene-gene interactions, the drought scenario strongly influences whether an allele will convey a significant advantage. In many cases, the effect of a trait on yield is a trade-off which depends on environmental conditions (Tardieu and Tuberosa, 2010). For instance, reduced transpiration and biomass accumulation protect against drought stress, yet reduce yield potential (Blum, 2009).

Modeling can be incorporated to address the challenges presented by gene-gene and gene-environment interactions in the physiological and genetic dissection of traits which convey drought tolerance, and ultimately in developing improved cultivars (Tardieu and Tuberosa, 2010). In addition to improving efficiency by dissecting complex traits into more measurable targets, which helps in the development of phenotyping strategies, using crop growth and development models to evaluate traits and predict phenotypes in the TPE is useful for assessing breeding strategies and allocating resources (Hammer et al., 2006; Messina et al., 2011). With the addition of environment modeling, the range of drought scenarios that comprise a TPE over time can be accounted for, helping to efficiently identify associations between traits and the set of environmental conditions which maximize yield.

Not only has modeling been used to enhance the physiological and genetic dissection of drought tolerance, it also has been used to augment a maize breeding program. By extending the concept of fitness landscapes to the characterization of yield-trait performance landscapes and extending the E (NK) model of trait genetic architecture to incorporate biophysical, physiological, and statistical components, Messina et al. (2011) developed a graphical representation of the associated yield-trait performance landscape that could be used in selection decisions. Doubled haploid (DH) lines were selected based on their relative position in the performance landscape, their predicted performance, and their potential to contribute to further yield improvement. This approach showed that an understanding of yield-trait performance landscapes can be used to improve genomic selection and phenotyping strategies.

Studies which use modeling have improved the predictive power of the combined effects of major QTL. In one study, a model based on the combined effects of the major QTLs was able to predict 74% of the variability for maize leaf elongation rate under a range of temperature and water deficit conditions (Reymond et al., 2003). Modeling has also been combined with phenotypic analysis to identify QTL for drought traits and to characterize genotypes (Tardieu, 2006). The development of response curves, which more quantitatively define the relationship between the phenotypic trait and environmental conditions, are unique to each genotype and can be compared to select optimal genotypes for a particular range of conditions. A similar approach has been used to identify common QTL for leaf growth and ASI under drought conditions, suggesting that the genetic determination of leaf growth and silk elongation rate is at least partially shared (Welcker et al., 2006). By extending the APSIM crop model to include genotype-specific parameters, Chenu et al. (2009) simulated the effects of QTL on leaf and silk elongation, and ultimately yield. The study demonstrated the high level of QTL × environment interaction, opening the possibility of exploiting these interactions for drought tolerance (Chenu et al., 2009).

### Marker assisted selection in breeding

While many drought tolerance candidate genes and QTL have been documented, few have been validated to produce a clear genetic gain when selected for in diverse germplasm under field conditions (Ribaut et al., 2009). This is in part due to many studies being limited to putative association based on colocalization of candidate genes and QTL along genetic maps. Another reason is that QTL or genes affecting drought tolerance are not distinguished based on how they are expressed under contrasting drought treatments. For breeding, QTL which are constitutively expressed and affect the yield consistently should be given priority, since they show limited interaction with the environment (Vargas et al., 2006). However, relatively simple heritable constitutive plant morphological and developmental traits are often ignored when evaluated by functional genomics, even though they have a considerable effect on performance under drought stress (Blum, 2011a). Other factors which limit the practical use of previous genetic investigations are the germplasm used for the mapping population, and the drought treatment used. Non-commercially viable lines are often used for association studies and identified QTL may have a null or negative effect when introgressed into elite materials (Monneveux and Ribaut, 2006). Regarding the drought treatment, many of the expressed genes identified in association studies are due to imposing rapid stress in a laboratory setting, but the slow progress of drought stress in field conditions results in minimal expression of these stress-responsive genes (Barker et al., 2005).

Although most information gathered from the genetic dissection of drought tolerance has not directly led to the development of improved cultivars, marker assisted selection has been used successfully. By introgressing favorable alleles at five genomic regions, MABC-derived hybrids were selected, which yielded 50% more than control hybrids under severe water stress conditions. Under mild stress and well-watered conditions, the hybrids performed as well as the control (Ribaut and Ragot, 2006). While the experiment did improve the recipient line through the introgression of drought QTL, further improvement is limited after the recurrent parent is fixed for the new QTL (Mir et al., 2012). To address this limitation, MARS has been used to take advantage of desirable alleles in multiple lines. The value of MARS in breeding for improved maize grain yield in SSA was demonstrated by Beyene et al. (2016) who estimated the rate of genetic gain under drought and non-drought environments. Since the value of a set of QTL alleles depends on the germplasm used in breeding, another MAS strategy was proposed by Podlich et al. (2004) to attempt to account for the presence of epistasis and G × E interaction. The “Mapping As You Go” (MAYG) strategy works by re-estimating the value of each QTL allele upon creation of a new set of germplasm, and its effectiveness has been estimated by simulation (Podlich et al., 2004).

Rather than selection based on known markers with significant associations to drought traits, the use of genomic selection (GS) or genome-wide selection (GWS) to develop drought tolerance promises improved gains (Bernardo and Yu, 2007). The application of GS in yield trials of tropical maize lines across multiple locations in SSA produced selection candidates at lower cost than phenotypic selection (Beyene et al., 2019). The cost aspect is important because in situations where doubled haploids (DH) lines are used, the capacity to phenotype testcross materials across multiple sites can limit progress (Beyene et al., 2021).

Genomic Selection described as “test-half-predict half” approach uses random markers to genotype a phenotyped training population. The marker and phenotyping data are used to develop breeding values of alternative alleles, which are fitted as random effects in a linear model. Selection in each recurrent generation is based on the sum of those breeding values, known as the genomic estimated breeding value (GEBV) (Meuwissen et al., 2001). This approach has considerable advantages, such as high selection accuracy when selecting on markers alone, and no prior knowledge of QTL positions is needed (Resende et al., 2014). The efficiency of the GS approach has been compared experimentally to selection based on yield alone along with selection incorporating secondary traits (Ziyomo and Bernardo, 2013). Compared to selection based on yield, secondary trait assisted selection was slightly higher or lower depending on the trait, while GS was significantly more efficient.

## Sources of genetic diversity

There are many sources of genetic diversity which potentially hold alleles promoting drought resistance (Barbosa et al., 2021). While conventional germplasm bases contain enormous levels of allelic polymorphism (Guo et al., 2004), the development of mutagenic and transgenic lines create a virtually endless supply of genetic diversity. Most sources of natural variation have remained relatively untapped. Globally, only 5% of the available maize germplasm is used commercially (Brown, 1975), and exotic germplasm constitutes only 1% of the US germplasm base (Goodman, 1983). This section aims to present resources that have been used or with potential in breeding for or dissecting drought tolerance.

An ideal germplasm base 1) segregates for the trait of interest, 2) has a high probability of containing desired alleles, and 3) those alleles should be relatively easy to introgress into cultivars (Blum, 2011b). The existing drought tolerance, genetic diversity, and ease of introgression make the agronomic germplasm pool an ideal first choice. Agronomic germplasm has been used to develop the Pioneer AQUAmax^®^ product line of drought-tolerant maize hybrids, improving upon the drought tolerance of commercial hybrids that already possess a high degree of tolerance (Cooper et al., 2014).

Alleles from landraces have been introgressed into elite varieties, leading to improved drought tolerance. Researchers at the International Institute of Tropical Agriculture (IITA) in Ibadan, Nigeria, crossed six landraces pre-screened for drought tolerance with an elite maize variety (AK9443-DMRSR), resulting in some of the BC_1_F_2_ populations expressing improved yield potential under random-drought conditions (Meseka et al., 2013). In dry, marginal growing regions of Kenya, farmers use local landraces rather than hybrids because they are believed to produce better under low or no input use (Sammons, 1987), and possess resistance to biotic and abiotic stress.

While wild germplasm and related species, such as Z*ea maxicana* or *Tripsacum floridanum*, are excellent sources of novel genes for drought tolerance improvement (Singh, 2010), there are some difficulties with their use. First, the technical difficulty in making a wide cross between an agronomic genotype and the donor. Second, the need to eliminate the introgression of negative traits carried by the donor, and third, the value of genes or alleles conveying drought resistance once introgressed (Blum, 2011b). Since adaptation to drought stress in wild type and related species enhances survival rather than optimizing yield, the genes which impart that resistance may not be beneficial when introgressed into breeding germplasm.

A molecular approach particularly suited to addressing this complication and identifying beneficial alleles from wild sources is advanced backcross QTL (AB-QTL) analysis (Tanksley and Nelson, 1996). After developing backcross families and eliminating lines displaying yield-reducing characteristics in the BC_1_ and BC_2_ generations, QTL analysis is performed. This approach has the benefit of identifying valuable QTL and developing superior genotypes simultaneously and has been used to analyze more conventional crosses as well (Ho et al., 2002).

### Transgenic and gene-editing tools

The application of mutagenesis techniques to improve drought tolerance in maize is limited (Blum, 2011b; Gao et al., 2014; Ruswandi et al., 2014). Therefore, transgenic approaches are useful in functional analysis of genes affecting stress response and adaptation (Yang et al., 2010), and can act as a bridge to move valuable genes into breeding germplasm (Blum, 2011b). Nelson et al. (2007) transformed plants to overexpress transcription factor *ZmNFYB2* resulting in increased drought tolerance and yield. Plants engineered to overexpress the *ZmAsr1* gene, a putative transcription factor, resulted in lines with improved water use efficiency and dry weight accumulation (Jeanneau et al., 2002). In another study, Nuccio et al. (2015) engineered plants to overexpress a gene encoding rice trehalose-6-phosphate phosphatase (*TPP*), which increased sucrose concentration in ear spikelets, kernel set, and harvest index, leading to improved yield in mild and severe drought. Guo et al. (2014) generated transgenic maize overexpressing the *ARGOS1* (*ZAR1*) gene resulting in enhanced maize organ growth, grain yield, and drought-stress tolerance.

While the transgenic approach has helped to identify genes and mechanisms that improve drought tolerance, there are obstacles to their use in released cultivars. Regarding transgenic events tested in laboratory conditions, the effect of the transgene might not yield an advantage in the TPE because of environmental interaction (Bänziger and Araus, 2007). When placed in agronomic germplasm, the expression of a single gene may not significantly alter the final phenotype due to dampening and compensation from other processes (Sinclair and Purcell, 2005). The mechanism of tolerance can also be dependent on the developmental stage (Flowers, 2004).

Despite these obstacles, there has been some success with the use of transgenic cultivars to mitigate the impact of drought stress on crops. One released cultivar developed using transgenics is the Droughtgard™ hybrids from Monsanto. By isolating and transferring a cold-shock protein gene (*cspB*) from the soil bacteria “*Bacillus subtilis*”, yield improvements from 11%–21% were achieved under drought conditions, with no negative effects under normal conditions. The protein cspB acts as a chaperone for other proteins and is believed to help disentangle RNA which has folded abnormally due to drought. Yield improvement due to the expression of *cspB* is primarily due to an increase in the number of kernels per plant (Castiglioni et al., 2008).

Novel genome editing tools, such as CRISPR-Cas9, have also been used to create genetic diversity resulting in improved drought response. Using CRIPR-Cas9, the native maize *GOS2* promoter was used by Shi et al. (2017) to both replace and supplement the native *ARGOS8* promoter. This created variants with altered expression of *ARGOS8*, a negative regulator of ethylene responses. Some variants achieved a yield gain of five bushels per acre under flowering stress conditions, with no yield penalty under well-watered conditions, demonstrating the viability of the editing tool (Shi et al., 2017). Gene knockout study of *ahb2* in maize *via* CRISPR/Cas9 resulted in quicker closure of stomata in response to water deficit stress, and three independent homozygous lines for the *i1*, *d2* and *d35* alleles that are tolerant to drought stress have been obtained (Liu et al., 2020). Gene editing approach will most likely be more attractive since crop varieties derived from this method could be considered non-genetically modified and thus be more acceptable.

## Breeding for maize drought tolerance in SSA

Decades of partnerships with public and private sector institutions by the International Maize and Wheat Improvement Center (CIMMYT) have resulted in successful breeding and deployment of elite stress-tolerant maize cultivars across SSA (Edmeades et al., 1996b; Prasanna et al., 2021). CIMMYT leveraged molecular tools such as QTL and MARS to improve maize tolerance to drought, as well as other important stresses such as nitrogen use efficiency, maize streak virus, and maize lethal necrosis (Semagn et al., 2015). Advances in molecular and systems biology provided new opportunities to accelerate the maize improvement progress in SSA (Wossen et al., 2017). As a result, the adoption of improved maize varieties tolerant to drought and other stresses has increased across the region (Chivasa et al., 2022).

Two projects that stand out in SSA are public-private funded collaborations: the Drought Tolerant Maize for Africa (DTMA) and Water Efficient Maize for Africa (WEMA) (Oikeh et al., 2014; Nasser et al., 2020). DTMA is implemented jointly by CIMMYT and the International Institute for Tropical Agriculture, in collaboration with national agricultural research systems in participating nations. WEMA is a partnership involving the Bill & Melinda Gates Foundation, USAID, the Howard G. Buffett Foundation, CIMMYT, Monsanto, the National Agricultural Research Systems (NARS) from the participating nations (Kenya, Uganda, Tanzania, South Africa, Ethiopia and Mozambique), and led by the African Agricultural Technology Foundation (AATF) a nonprofit organization. The projects used conventional breeding, double haploid technology (DHT), transgenic technology, and marker assisted breeding. The objectives of WEMA and DTMA projects were to develop and distribute drought tolerant white maize hybrids that would yield more under drought stress than commercially used varieties (Oikeh et al., 2014; Nasser et al., 2020).

In the DTMA project, researchers used MARS to improve locally adapted germplasm and identify genomic regions associated with drought tolerance. The DTMA project resulted in the registration of 160 drought tolerant maize hybrids for release across 13 SSA countries between 2007 and 2013, with an adoption rate of between 9%–61% in six countries (Fisher et al., 2015). In 2020, 27 new, multiple-stress-tolerant maize hybrids and open-pollinated varieties were released by small and medium sized enterprises and national agricultural research system (NARS) partners for commercialization in SSA.

In the WEMA project, lines used to create the hybrids were also developed using DHT and selection achieved through MARS (Edge et al., 2017). About 106 non-transgenic drought tolerant maize hybrids were released across five participating countries (Edge et al., 2017), and sold under the name, DroughtTEGO, as well as several genetically modified (GM) varieties marketed under the brand name TELA, which are both drought tolerant and insect resistant from the Bt gene (Oikeh et al., 2014; Edge et al., 2017).

## Plant breeding education

The shortage of plant breeders in SSA presents a serious challenge because it limits the development of improved varieties of crops for regional food security (Suza et al., 2016). To sustain the success of the maize drought tolerance projects, a pipeline of plant breeders and crop scientists must be in place in SSA. Support for human capacity enhancement can be modeled using successful partnerships such as Improved Master of Science in Cultivar Development for Africa and Plant Breeding E-Learning in Africa (Suza et al., 2016). In addition, centers in Africa, such as the African Centre for Crop Improvement (ACCI) in South Africa and West Africa Centre for Crop Improvement (WACCI) in Ghana, with funding largely from the Alliance for a Green Revolution in Africa (AGRA) have contributed plant breeding capacity in Africa. Several of the breeders from ACCI and WACCI have identified germplasm that contain useful alleles for drought tolerance improvement of maize inbred lines and hybrids under drought stress (Derera et al., 2008; Adebayo and Menkir, 2015; Meseka et al., 2018; Nasser et al., 2020). Evaluation of these germplasm has been achieved mainly through the utilization of conventional breeding *via* indirect selection using secondary traits drought selection indices. Other studies involving plant breeders from ACCI and WACCI also utilized marker-assisted recurrent selection and reported genetic gain under drought stress ranging from 22.7 kg ha^−1^ yr^−1^–118 kg ha^−1^ yr^−1^ (Beyene et al., 2016; Bankole et al., 2017; Masuka et al., 2017).

## Conclusion

Since the development of hybrid maize, progress in breeding for yield potential has consistently improved drought tolerance. However, new strategies are needed to address the increase in demand and challenges brought about by climate change. While the goal is improved yield under drought conditions and yield stability, selecting on yield alone is inefficient due to the low heritability under stress conditions. Physiological dissection of yield into more heritable and selectable secondary traits can lead to better phenotyping strategies and improved gains. Advances in molecular biology provide new tools to understand the genetic basis of drought tolerance. Such tools have helped to identify associations between QTL and drought tolerance, yet gene interactions such as epistasis and pleiotropy, and gene by environment interactions complicate the use of genomic information for breeding. Integrating molecular genetics with physiology will help untangle the complex network of gene and environmental interactions, promoting the identification of the most promising loci conveying drought tolerance. Ecophysiological modeling can be used to better understand how genetic variability translates into the final phenotype by incorporating genomic information with known principles of crop growth and development. By integrating the vast amount of environmental, genetic, and physiological knowledge and applying it to a well-chosen germplasm base, plant breeders will be able to meet the challenge of drought stress and continue to deliver improved varieties in the future. The DTMA and WEMA projects in SSA emphasize the importance of multidisciplinary approaches that incorporate multiple breeding tools and approaches In addition, financial investment in human and institutional infrastructure capacity strengthening are needed to sustain the application and adoption innovations such as DTMA and WEMA in Africa.
